# Ideal Dentistry

**Published:** 1880-04

**Authors:** C. T. Stockwell

**Affiliations:** Springfield, Mass.


ARTICLE III.

### IDEAL DENTISTRY.

BY C. T. STOCKWELL, SPRINGFIELD, MASS.

Upon the library walls of St. Andrew's, Scotland's most ancient university, is painted this motto from the old poet Homer, viz: "Always strive for the best." Instead of this, perhaps, an interpreter of our day would have rendered it, "Always strive for the highest."

The product of the subtle force and power of this simple motto, standing there for nearly five centuries past, looking down upon the thousands of students as they continued their studies for a series of years during the formative period of their lives, is beyond our imagination to compute. Could they have failed to absorb more or less the inspiration of these words? Many of them, it is said, have in after life remarked that they were indebted, more than anything else, for their success to the impulse developed in their university days as the result of that influence which was almost unconsciously but continuously distilled upon them by Homer's "Always strive for the best." This result not only proves the sagacity of the old founders of St. Andrew's, but is also a notable instance of a recognition of the fact that a high ideal is the prime power which ultimates in real success.

The thought that I wish to briefly consider is the importance or need we have of an ideal, and that by the consideration of the ideal we may measure the distance between the real and the ideal. We need an ideal to insure any progress at all. We need it to arouse within us a discontent for present attainments. We must, at least, catch a glimpse of something better before it is possible for us to aspire even to be or do better. This applies to the whole realm of life, moral, intellectual and professional. An ever-present consciousness of the ideal is the great incentive, almost the only power that impels an effort, or that kind and quality of an effort which results in real progress. Suppose we apply this thought for a moment to society. We look around us upon the real and see much of suffering, sorrow, wrong, misery and corruption in all its forms, mingled with that which is wiser, better, happier, and purer in a greater or less degree; and then if we lift the mind to a conception of a true ideal of what society as a whole might be, we perceive that a vast distance stretches between the real and ideal society.

Again, by picturing in our minds a perfect character, or taking as our ideal some known character, and considering it until its completeness and fullness, its beauty and loveliness, its grandeur and desireableness have rounded and filled our ideal in all its parts to a perfect conception; and then withdraw our eyes from that grand height, letting them rest upon our real selves, I think we shall see that by the use of the ideal we may discover our distance from perfection; and not only this, but that we shall, by a consciousness of this fact, be made discontented with ourselves, which condition of the mind may, perhaps, be rightly considered the parent of the impulse or inspiration that induces us to "strive for the best," or highest.

This conception of things in their most perfect state we need, I think, as dentists, to dwell more upon. The cultivation of the ideal in our specialty I regard an indispensable factor in our progress therein; and the ratio of our advancement will be proportionate with the cultivation of this element. Thus far no ground, I apprehend, for a diversity of opinions is taken; but by venturing to answer the question,

"What, then, is the ideal dentistry?" difficulties arise, and views will likely be disputed. To some the answer would seem to be an ideal artificial denture; to others an ideal filling material, or an ideal manner of manipulation of some material. While to still others, and a rapidly growing class, a better answer would perhaps be this: The ideal dentistry is that which in its results accomplishes the most in doing away with the need of dentists at all. In other words, the ideal dentistry is preventive rather than curative.

This should be the high aim of every member of the profession. No one should hesitate to extend his influence to all measures that are calculated to accomplish this purpose for fear of killing the goose that lays such golden eggs.

The high calling of the dentist is to teach the public what these preventive measures are. The repairing of broken-down tissue, or the substitution of artificial dentures, each has its respective ideals, and should be zealously prosecuted; but after all they should be secondary and accidental. The climax in dentistry lies beyond. In our aspirations and ambitions to seek out the ideal material for fillings and ideal methods of manipulation, we too often seem to overlook the greater importance of seeking out preventive measures.

If by some chance a life-long patient of some noted dentist falls into our hands, and we find the teeth all ablaze with glittering fillings, we at once find ourselves admiring the remarkable skill and proficiency of the said noted dentist. We perhaps do not even think of the fact that, possibly, all of this glitter is, after all, but an impeachment of the dentist.

Another case comes to us, where the patient has from early childhood been under the constant care of Dr. ——. We find the patient, still in middle life, wearing a beautiful set of artificial teeth, answering in every particular, if you please, to the ideal plate. Again we find ourselves admiring the skill and ingenuity of the popular dentist; and we also find the patient loud in his praise. The patient and ourselves should rather, in at least very many of these cases, see that this beautiful plate of artificial teeth, ideal though it be, is but the "handwriting upon the wall," the self-evident testimony to the fact that the dentist has been "weighed in the balance and found wanting" in that which constitutes the highest ability and skill. The highest place should be given to that dentist whose patients of long standing exhibit the fewest fillings and artificial substitutes.

Accepting this view as the correct answer to our question we must perceive that the ideal and real are wide apart, and that a vast field stretches away before us for investigation and research beyond mere theories and speculations of causes. Supposing the electric action does augment the chemical in secondary decay, and supposing the chemical action is alone chargeable in primary decay, or suppose it is the vital-chemical, the fact still remains that the great devil we have to contend with is the acids.

Can he be outwitted or overthrown by merely mechanical skill, however perfect? or when he has once laid siege and broken down our walls, can the breaches be replaced by any material that will withstand his renewed attacks, unless he is utterly routed and the conditions changed so as to render his presence improbable? Our patients need to be more thoroughly educated regarding the absolute necessity of properly cleansing their teeth, and the object and use of antacid agents; and the dentist may find a fruitful field for investigation and research in constitutional conditions and treatment relating to his specialty. If it be a fact that a large proportion of these destructive acids have their origin in abnormal constitutional conditions, constitutional treatment would seem to be indicated as within the legitimate province of the dental practitioner, and should not be considered an encroachment upon the medical practitioner's domain. Preventive treatment in order to accomplish its object must embrace constitutional treatment; and we, as a profession, will fall short of reaching the true ideal unless we can successfully claim and occupy this field.

